# Study on the relationship between DNA methylation of target CpG sites in peripheral blood and gestational diabetes during early pregnancy

**DOI:** 10.1038/s41598-021-99836-2

**Published:** 2021-10-14

**Authors:** Xiaolei Wang, Jin Huang, Yixiang Zheng, Sisi Long, Huijun Lin, Na Zhang, Mengyuan Tian, Xinrui Wu, Rongjing An, Shujuan Ma, Hongzhuan Tan

**Affiliations:** 1 Department of Epidemiology and Health Statistics, Xiangya School of Public Health, Central South University, Xiangya Road, Kaifu District, Changsha City, Hunan Province 410078 China; 2 Reproductive and Genetic Hospital of CITIC-Xiangya, Clinical Research Center for Reproduction and Genetics in Hunan Province, Changsha City, Hunan Province 410008 China; 3 Hospital Infection Control Center, The Second Xiangya Hospital, Central South University, Changsha City, Hunan Province 410078 China; 4 Hunan Provincial Key Laboratory of Clinical Epidemiology, Changsha City, Hunan Province 410078 China; 5 Department of Infectious Diseases, Key Laboratory of Viral Hepatitis of Hunan, Xiangya Hospital, Central South University, Changsha City, Hunan Province 410078 China

**Keywords:** Epigenetics, Gestational diabetes

## Abstract

Genome-wide DNA methylation profiling have been used to find maternal CpG sites related to the occurrence of gestational diabetes mellitus (GDM). However, none of these differential sites found has been verified in a larger sample. Here, our aim was to evaluate whether first trimester changes in target CpG sites in the peripheral blood of pregnancy women predict subsequent development of GDM. This nested case–control study was based upon an early pregnancy follow-up cohort (ChiCTR1900020652). Target CpG sites were extracted from related published literature and bioinformatics analysis. The DNA methylation levels at 337 CpG sites of 80 GDM cases and 80 matched healthy controls during the early pregnancy (10–15 weeks) were assessed using MethylTarget sequencing. The best cut-off level for methylation of CpG site was determined using the generated ROC curve. The independent effect of CpG site methylation status on GDM was analyzed using conditional logistic regression. Methylation levels at 6 CpG sites were significantly higher in the GDM group than in controls, whereas those at another 6 CpG sites were significantly lower (FDR < 0.05). The area under the ROC curve at each methylation level of the significant CpG sites ranged between 0.593 and 0.650 for the occurrence of GDM. After adjusting for possible confounders, the hypermethylation status of CpG site 68167324 (OR = 3.168, 1.038–9.666) and 24837915 (OR = 5.232, 1.659–16.506) was identified as more strongly associated with GDM; meanwhile, the hypermethylation of CpG site 157130156 (OR = 0.361, 0.135–0.966) and 89438648 (OR = 0.206, 0.065–0.655) might indicate lower risk of GDM. The methylation status of target CpG sites in the peripheral blood of pregnant women during the first trimester may be associated with GDM pathogenesis, and has potential as a predictor of GDM.

## Introduction

Gestational diabetes mellitus (GDM) is defined as any degree of glucose intolerance with onset or first recognition during pregnancy^1^. Alongside the increasing prevalence of obesity and type 2 diabetes, the incidence of GDM has risen annually^2^. Specifically, in China, this prevalence has been estimated at 14.8% (12.8–16.7%)^3^. GDM in pregnant women can result in severe adverse pregnancy outcomes complications, including macrosomia, premature birth, and fetal malformations^4^. GDM also has serious consequences for the offspring, such as childhood obesity, insulin resistance^5^, and impaired neurodevelopment^6^. In addition, GDM can recur in future pregnancies and also increases risk for postpartum type 2 diabetes^7^.

The mechanisms underlying GDM include but not limit to genetic background^8^, inflammatory factors^9^, and oxidative stress^10^. Wu et al. have shown that GDM has a genetic component, and the differences in GDM among ethnicity may be due to the interaction between genes and the environment^11^. Epigenetics bridges the gap between genes and the environment at the molecular level^12,13^. DNA methylation is one of the most commonly studied epigenetic modifications, which is catalyzed by DNA methyltransferases and uses S-adenosylmethionine as the methyl donor to convert the CpG site dinucleotide cytosine to the 5′-methylcytosine. The CpG site density and methylation degree of the upstream promoter region of the gene directly affect gene activity and expression^13^. DNA methylation therefore plays a key role in regulating genome transcription. Emerging studies from placental tissue and fetal cord blood suggest that in utero exposure to GDM impacts the placental and fetal epigenome^8,14,15^. However, these non-invasive samples collected at delivery during childbirth cannot reflect the level of maternal methylation before the onset of GDM, and could not be used for the exploration of the pathogenesis of GDM by methylation.

Peripheral blood samples of pregnant women can be used to reflect changes in DNA methylation levels during pregnancy. Current researches focused on GDM and DNA methylation are mainly based on the genome-wide DNA methylation analysis^16,17^. Kang et al. conducted genome-wide DNA methylation chip analysis of the peripheral blood samples in late pregnancy and revealed that the methylation levels of 200 CpG sites of 151 genes are different between the eight GDM patients and eight healthy controls^18,19^. Moreover, Wu et al. observed that the methylation levels of 100 CpG sites corresponding to 66 genes were different between the GDM group (n = 11) and the control group (n = 11), in the peripheral blood of pregnant women in their first trimester, further suggesting that the DNA methylation status of 5 CpG sites, in the *COPS8, PIK3R5, HAAO, CCDC124,* and *C5orf34* genes could be used as clinical biomarkers of GDM^17^. Similarly, Enquobahrie et al.^20^ found 17 hypomethylated and 10 hypermethylated CpG sites in the GDM group with the help of genome-wide DNA methylation analysis.

Although genome-wide DNA methylation scanning can broadly mine differentially methylated CpG sites as potential candidate genes for disease diagnosis and prediction, prior studies on this topic have been limited by small sample sizes, ethnic differences, method of quantification and lack of verification^16^, the reproducibility of the test is poor, and the difference sites found among studies are less overlapping. On the contrary, the differential DNA methylation detection of candidate genes is a targeted identification based on existing discoveries and potential mechanisms. Here, we evaluated the DNA methylation levels in the peripheral blood of women in early pregnancy using specific target gene DNA methylation detection in order to verify the relationship between the methylation status of the targeted CpG sites and the onset of GDM.

## Material and methods

### Study design and population

This was a nested case–control study based on an early pregnancy follow-up cohort. The cohort was established in Hunan Province Maternal and Child Health Hospital (ChiCTR1900020652) between March 2017 and December 2018, and a total of 890 pregnant women were enrolled. All the eligible participants agreed to participate in this study and provided written informed consent. The study protocol was approved by the Medical Ethical Committee of the Hunan Provincial Maternal and Child Health Hospital in South China (approval number: EC201624 on January 11, 2017) and all methods were performed in accordance with the relevant guidelines and regulations. Pregnant women were recruited in their first trimester (10–14 weeks) and followed up for 42 days post-partum. The inclusion criteria were: (1) singleton pregnancy and natural conception; (2) diabetes-free at recruitment; (3) had not received any antibiotic treatment throughout the current pregnancy; (4) no acute infection in the 2 weeks before sample collection; (5) planned to attend for all obstetric examinations and delivery at the above hospital. We collected questionnaire data and venous blood samples, while additional patient information and data concerning their clinical examinations were collected through the hospital’s electronic recodes system. The venous blood samples (5 ml/person) were collected using blood collection tubes without anticoagulant during early pregnancy (10–15 gestational weeks), by certified nurses in the morning following a 10-h overnight fast. Serum and blood cells were separated by centrifugation at 3500 rpm for 15 min and stored at − 80 °C until further use.

### Diagnostic criteria for GDM and selection of controls

All subjects underwent a 2-h standard 75 g oral glucose tolerance (OGTT)^21^ in the hospital outpatient department at 24–28 weeks of gestation. The oxidase method was used to estimate blood glucose levels, with measurement completed using an automatic biochemical analyzer (Hitachi 7600) at the hospital. GDM was defined according to the International Association of Diabetes and Pregnancy Study Groups (IADPSG) standard. That is, GDM was considered to be present when at least one of the following blood glucose concentrations was obtained: ≥ 5.1 mmol/L (fasting), ≥ 10.0 mmol/L (after 1 h), and ≥ 8.5 mmol/L (after 2 h)^21^. The controls were selected from women in the same cohort who had normal blood glucose levels throughout the pregnancy. A 1:1 pair match for each GDM patient was identified, based on the age of the pregnant (± 3 years) and gestational week (± 1 week) at the time of enrollment, resulting in a final study population of 80 eligible GDM patients and 80 healthy controls.

### Selection of candidate CpG sites

Candidate CpG sites were mainly selected from published studies, and supplemented by bioinformatics analyses (Supplementary Table S3). Through a systematic review^17–20,22^, 21 target CpG sites were collected from the differential loci found in previous GDM-related whole-genome methylation sequencing analysis literatures, another 6 CpG islands were generated from the promoter regions of four genes which were reported to be closely related to the pathogenesis of GDM^17,23,24^. Additionally, two target sites were identified from the methylation data of cervical cancer patient data sets in the The Cancer Genome Atlas (TCGA) and gene expression omnibus (GEO) databases by T-test method, with the Q value obtained from Benjamini and Hochberg modified *P* value controlling the false discovery rate of multiple hypothesis testing. Through the target site, the 50–100 bp upstream or downstream of its location was selected as the sequenced fragment, and all the CpG sites in the fragment were sequenced. Overall, this procedure therefore identified a total of 29 target fragments, containing 337 CpG sites. The primers and their sources are shown in Supplementary Tables S1, S2.

### DNA extraction

Genomic DNA was extracted from frozen samples using Genomic Tip-500 columns (Qiagen, Valencia, CA, USA) and from bisulfite-converted samples using the EZ DNA Methylation™-GOLD Kit (Zymo Research, CA, USA) in accordance with the manufacturer’s instructions. Genomic DNA integrity was measured using agarose gel electrophoresis and quality control was ensured using a NanoDrop 2000 (NanoDrop technologies, Wilmington, DE, USA), which requires that the DNA concentration ≥ 20 ng/μL, and that the total amount of DNA ≥ 1 μg.

### DNA methylation analysis

The DNA methylation level of the target CpG site is defined as the number of methylated reads at that site (i.e., the number of reads with base C detected) divided by the total number of reads at that site, and was obtained by MethylTarget sequencing (Genesky Biotechnologies Inc. Shanghai, China), a method based on next-generation sequencing-based multiple targeted CpG methylation analysis. Primer design and validation were performed using bisulfate-converted DNA samples on the Methylation Primer software. The primer sets were designed to flank each target CpG site by 100–300 nucleotides and are summarized in Supplementary Table S1. After PCR amplification (HotStarTaq polymerase kit, TAKARA, Tokyo, Japan) and library construction, paired-end sequencing was performed (Illumina Hiseq Benchtop Sequencer, CA, USA) in accordance with the manufacturer’s protocol.

### Quality control

In order to ensure the consistency of the DNA methylation level detection results of all CpG sites, all DNA samples were sent in the same batch and tested using the same test method. For the data results after sequencing, we evaluated the quality of the original data through Fast QC software. The main evaluation index was the basic quality index (Q value). During sequencing, Q20 indicates that the Q value is greater than or equal to 20, that is, the sequencing error rate (P) during sequencing is less than or equal to 1%; Q30 indicates that the Q value is greater than or equal to 30, that is, the sequencing error rate (P) during sequencing is less than or equal to 0.1%. Generally, Q20 ≥ 90% (ie 90% base sequencing error rate ≤ 1%), and Q30 ≥ 85% (ie 85% base sequencing error rate ≤ 0.1%) are considered as qualified sequencing results. At the same time, the lowest conversion rate of bisulfite in the DNA methylation level detection process was 98.88%. For details, see Supplementary Data QC (excel file).

### Covariates

In this study, we collected information on maternal demographics, lifestyle, and pregnancy history through structured questionnaires during each follow-up. This included factors with the potential to confound the exposure-outcome relationship, including pre-pregnancy body mass index (BMI) (continuous), history of drinking (yes/no, defined as drinking alcohol one or more times for 6 consecutive months), history of smoking (yes/no), parity (continuous), pregnancy order (continuous), polycystic ovary syndrome (PCOS) (yes/no), and waist circumference at enrolment (continuous). Many included participants were primiparas, so history of GDM was not considered in the analysis.

### Statistical analysis

Continuous data and categorical data were represented by the mean ± standard deviation (SD) and frequency (percentage), respectively. Paired-samples T test were used to compare normally distributed continuous data, whereas Wilcoxon signed rank test were used to analyze non-normally distributed continuous data. Meanwhile, FDR (False positive rate) correction analysis was performed for CpG sites with differences in univariate analysis. Dichotomous variables were analyzed using McNemar χ^2^ test. *P* < 0.05 was considered statistically significant, and all statistical tests were two-sided. ROC curve analysis was used to assess the possible predictive value of the methylation level of individual CpG site for the occurrence of GDM. When the level of DNA methylation was positively correlated with GDM, GDM would be used as the value of the state variable for ROC curve analysis, conversely, the control would be used. Through the ROC curve, the methylation status (high or low) of the target CpG site was classified based on the best cut-off value, defined as the DNA methylation level with the highest Youden index. Conditional logistic regression analysis was used to determine the independent influence of target CpG site methylation status on GDM. The model variable selection criterion was α_in_ = 0.05; the variable elimination criterion was α_out_ = 0.10; the Wald forward method was used to establish a conditional logistic regression model to screen CpG sites with independent effects. All the statistical analyses were performed using SPSS software v25.0 (IBM Corporation, Armonk, NY, USA).

## Results

### Patient characteristics

The participant characteristics are summarized in Table 1. The age of the GDM patients ranged between 23 and 43 years (mean: 31.6 years), whereas that of the healthy controls ranged between 24 and 45 years (mean: 32.0 years). No significant difference (*P* > 0.05) was observed between the two groups in terms of gravidity, parity, PCOS, smoking history, alcohol intake history, age, or gestational age. The GDM group had higher fasting glucose, 1-h post-OGTT glucose, 2-h post-OGTT glucose, pre-pregnant BMI, and waist circumference than the control group.

**Table 1:** Study participant’s characteristics.

Variable	Control (N = 80)		GDM (N = 80)		*P* value
**Participant**					
Gravidity, M (Q1–Q3)	2 (1–3)		2 (1–3)		0.750
Parity, M (Q1–Q3)	1 (0–1)		1 (0–1)		0.950
PCOS, n (%)	2 (2.5)		7 (8.8)		0.086
Smoking history, n (%)	2 (2.5)		1 (1.3)		0.560
Drink history, n (%)	3 (3.8)		3 (3.8)		1
**Demographic characteristics, Mean ± SD, range**					
Maternal age (years)	31.6 ± 4.3	23.0–43.0	32.0 ± 4.5	24.0–45.0	0.617
Gestational age (weeks)	12.7 ± 0.7	10.0–14.4	12.6 ± 0.8	9.9–14.3	0.634
Pre-pregnant BMI (kg/m^2^)	20.4 ± 2.6	16.2–30.9	22.4 ± 3.2	16.7–31.9	< 0.001
Waist circumference (cm)	77.3 ± 7.5	63.0–108.0	82.0 ± 9.2	58.0–107.5	< 0.001
**75 g OGTT in second trimester, Mean ± SD, range**					
Fasting glucose (mmol/L)	4.4 ± 0.3	3.8–5.0	4.9 ± 0.5	3.9–5.9	< 0.001
1-h post-OGTT (mmol/L)	6.4 ± 0.9	3.5–7.7	9.3 ± 1.6	5.0–13.0	< 0.001
2-h post-OGTT (mmol/L)	5.7 ± 0.6	3.8–6.6	8.2 ± 1.4	4.0–11.3	< 0.001

Abbreviation: *M* median; (Q1–Q3), interquartile ranges, *PCOS* polycystic ovary syndrome.

### Varying DNA methylation levels at target CpG sites

We assessed the DNA methylation levels at 337 CpG sites, and the results of the paired-samples T test or Wilcoxon signed rank test analysis for all the CpG sites are summarized in Supplementary Table S4. The mean imputation method was used to deal with missing data (Supplementary Table S4). There were 13 CpG sites with results differing significantly between the group (Table 2; scatter diagram in Supplementary Figure S1). Through FDR correction analysis, the methylation levels at 6 CpG sites within the *ARHGAP40, STAT1, C5orf34, RDH12,* and *YAP1* genes were higher in the GDM group than in the control group, whereas those at 6 CpG sites within the *HAPLN3, IFNGR2, YAP1, NFATC4,* and *DNAJB6* genes were lower in the GDM group than in the control group. Brief introduction to the function of the genes where those differential CpG sites are located can be found in Supplementary Table S5.

**Table 2:** Varying DNA methylation target CpG site and related genes.

CpG site ^a^	Gene Symbol	Chr	Distance to TSS	Methylation level (Mean ± SD)		difference ^b^	*P* value	FDR
				GDM (80)	Control (80)			
chr20: 37274257	ARHGAP40	20	( +) 43,681	0.601 ± 0.046	0.584 ± 0.048	0.017	0.015	0.033
chr5: 43487508	C5orf34	5	( +) 27,765	0.456 ± 0.078	0.414 ± 0.105	0.042	0.012	0.044
chr7: 157130156	DNAJB6	7	( +) 446	0.010 ± 0.006	0.012 ± 0.005	0.002	0.013	0.033
chr7: 157130085	DNAJB6	7	( +) 375	0.006 ± 0.003	0.007 ± 0.004	0.001	0.018	0.031
chr11: 101980999	YAP1	11	( −) 192	0.009 ± 0.003	0.007 ± 0.003	0.002	0.001	0.013
chr11: 101981060	YAP1	11	( −) 131	0.011 ± 0.004	0.012 ± 0.004	0.001	0.028	0.036
chr21: 34775358	IFNGR2	21	( +) 157	0.024 ± 0.038	0.036 ± 0.039	0.012	0.012	0.044
chr22: 42466321	NAGA	22	( +) 525	0.049 ± 0.014	0.055 ± 0.016	0.006	0.008	0.052
chr14: 68167324	RDH12	14	( −) 1278	0.273 ± 0.009	0.270 ± 0.009	0.003	0.019	0.027
chr14: 68167386	RDH12	14	( −) 1216	0.314 ± 0.009	0.311 ± 0.013	0.003	0.042	0.045
chr15: 89438648	HAPLN3	15	( +) 209	0.011 ± 0.004	0.013 ± 0.006	0.002	0.048	0.048
chr14: 24837915	NFATC4	14	( −) 288	0.015 ± 0.003	0.014 ± 0.003	0.001	0.018	0.031
chr2: 191879104	STAT1	2	( −) 128	0.008 ± 0.003	0.009 ± 0.005	0.001	0.037	0.043

Abbreviation: *Chr* chromosome, *TSS* transcription start site and strand; CpG site ^a^: position on chromosome; difference ^b^: the difference of the mean methylation level in the GDM group to that in the Control group.

### Roc curve analysis of different sites

For the 12 significantly different DNA methylation CpG sites, we further estimated the possible predictive value of the methylation level of individual CpG site for the occurrence of GDM using the ROC curve. The ROC curve parameters and the cut-off value are summarized in Table 3. The largest area under the curve (AUC) reached 0.650.

**Table 3:** The AUC and cut-off value of CpG site DNA methylation.

CpG site	Gene Symbol	Cut-off value	Sensitivity	Specificity	AUC	95% CI	*P* value
**For GDM**							
chr20: 37274257	ARHGAP40	0.600	0.550	0.638	0.594	0.506–0.682	0.040*
chr5: 43487508	C5orf34	0.420	0.775	0.475	0.602	0.514–0.691	0.026*
chr14: 24837915	NFATC4	0.014	0.713	0.525	0.628	0.541–0.715	0.005*
chr14: 68167386	RDH12	0.315	0.475	0.750	0.601	0.513–0.689	0.028*
chr14: 68167324	RDH 12	0.278	0.550	0.563	0.589	0.501–0.677	0.051
chr11: 101980999	YAP1	0.009	0.525	0.713	0.650	0.566–0.735	0.001*
**For Control**							
chr15: 89438648	HAPLN3	0.013	0.688	0.532	0.597	0.509–0.688	0.034*
chr7: 157130156	DNAJB6	0.013	0.747	0.463	0.608	0.521–0.696	0.019*
chr2: 191879104	STAT1	0.008	0.663	0.525	0.583	0.495–0.672	0.069
chr7: 157130085	DNAJB6	0.004	0.506	0.688	0.593	0.505–0.681	0.043*
chr11: 101981060	YAP1	0.014	0.550	0.575	0.582	0.494–0.670	0.074
chr21: 34775358	IFNGR2	0.028	0.671	0.566	0.614	0.533–0.708	0.014*

*P < 0.05.

### Comparison of the DNA methylation status of target CpG sites

To clearly show the effect of DNA methylation at the target CpG sites on GDM occurrence, we classified the DNA methylation levels into hypermethylation and hypomethylation statuses based on the best cut-off value (Table 3). Table 4 presents the differences in the DNA methylation statuses of the CpG sites between the GDM and control groups. Significant differences were observed in 8 CpG sites based on the McNemar χ^2^ test (*P* < 0.05).

**Table 4:** Distribution of DNA methylation at CpG sites between GDM group and control group.

CpG site	Gene symbol	GDM (n)		Control (n)		χ^2^	*P* value
		hypo-	hyper-	hypo-	hyper-		
chr20: 37274257	ARHGAP40	38	42	51	29	1.21	0.328
chr15: 89438648	HAPLN3	61	19	42	38	5.34	0.027*
chr14: 24837915	NFATC4	23	57	40	40	7.57	0.043*
chr5: 43487508	C5orf34	16	64	36	44	4.59	0.000*
chr21: 34775358	IFNGR2	55	25	33	47	0.63	0.488
chr14: 68167324	RDH12	55	25	69	11	29.33	0.000*
chr14: 68167386	RDH12	42	38	59	21	7.00	0.011*
chr11: 101980999	YAP1	38	42	57	23	3.69	0.072
chr11: 101981060	YAP1	65	15	56	24	18.89	0.000*
chr7: 157130156	DNAJB6	46	34	62	18	12.25	0.001*
chr2: 191879104	STAT1	42	38	27	53	1.27	0.305
chr7: 157130085	DNAJB6	25	55	16	64	17.09	0.000*

*P < 0.05; hypo-, hypomethylation; hyper-, hypermethylation.

### Conditional logistic regression analysis for DNA methylation status and GDM

Conditional logistic regression analysis was used to analyze the independent effect of the methylation status of the individual site on GDM occurrence. The independent variables included the methylation status of the eight significantly different CpG sites listed in Table 4 (0 = “hypomethylation”; 1 = “hypermethylation”). The confounding variable included waist circumference and pre-pregnancy BMI.

We found that the methylation status of four CpG sites influenced GDM occurrence. Specifically, the hypermethylation of CpG site 68167324 (OR = 3.168; 95% CI 1.038–9.666), and CpG site 24837915 (OR = 5.232; 95% CI 1.659–116.506) may indicate increased risk of GDM occurrence. In contrast, the hypermethylation of CpG site 157130156 (OR = 0.361; 95% CI 0.135–0.966) and CpG site 89438648 (OR = 0.206; 95% CI 0.065–0.655) may indicate decreased risk of GDM (Table 5).

**Table 5:** Conditional logistic analysis of methylation status of CpG sites on GDM.

CpG site	Gene symbol	β	Wals 2	*P* value	OR	95% CI
chr15: 89438648	HAPLN3	− 1.581	7.164	0.007	0.206	0.065–0.655
chr14: 24837915	NFATC4	1.655	7.970	0.005	5.232	1.659–16.506
chr14: 68167324	RDH12	1.153	4.104	0.043	3.168	1.038–9.666
chr7: 157130156	DNAJB6	− 1.020	4.116	0.042	0.361	0.135–0.966

*OR* odds ratio, *β* regression coefficients, *95% CI* 95% confidence interval of OR.

## Discussion

An increasing number of studies have explored the pathogenesis of GDM from the perspective of epigenetics. However, most of these were small (< 30 GDM cases), and they mainly observed the associations between GDM occurrence and the DNA methylation level of cord blood or placental tissue^16,25–27^. In this study, we evaluated the DNA methylation status of GDM-related CpG sites in the peripheral blood of women in early pregnancy using MethylTarget sequencing. In addition, we verified the associations between target CpG sites and GDM using relatively large sample size (80 GDM cases and 80 matched controls). Overall, we identified 13 CpG sites with significant differences in DNA methylation levels between the GDM and control groups based on quantitative analysis. The AUCs of the ROC curve for each methylation level of the significant CpG sites ranged from 0.593 to 0.650 predictive utility in relation to GDM. The methylation status of eight individual CpG sites were identified as differing significantly between GDM and control groups by qualitative analysis, and these were located in the promoter regions of *RDH 12, HAPLN3, NFATC4, YAP1,* and *DNAJB6,* and the intron region of *C5orf34*. Importantly, we found that the methylation statuses of four CpG sites were significantly associated with GDM occurrence, namely CpG site 89438648 (*HAPLN3*), 68167324 (*RDH12*), 157130156 (*DNAJB6*), and 24837915 (*NFATC4*), using conditional logistic regression analysis.

In this study, hypermethylation of the CpG site 89438648, located in the promoter region of *HAPLN3*, was found to suggest a lower risk of GDM (OR = 0.206; 95% CI 0.065–0.655). *HAPLN3* codes for hyaluronan and proteoglycan link protein 3 (HAPLN3), and the connexin 3 belong to the hyaluronic acid and proteoglycan connexin (HAPLN) family, which plays roles in the aggregation of proteoglycans and hyaluronic acid, and in cell adhesion^28^. HAPLN3 is involved in the organization and stability of the hyaluronic acid (HA)-dependent extracellular matrix (ECM) in many tissues. HA is one component of the ECM within the islet tissue of humans and mice^29^. It can cause islet amyloid deposition, which is associated with decreased β-cell area and an increase in β cell apoptosis^30^. Hull et al. suggested that islet amyloid deposition could reduce the number of β-cells^30,31^. Hypermethylation of the CpG site 89438648 located in the HAPLN3 promoter region, could reduce the level of HAPLN3, in turn reducing the stability of the HA-ECM, and consequently reducing the impact amyloid deposition on β cells.

We found that the hypermethylation status of CpG site 68167324 located in *RDH 12*, can increase the risk of GDM (OR = 3.168; 95% CI 1.038–9.666). *RDH 12* encodes retinol dehydrogenase 12 (RDH12), a member of the short-chain dehydrogenases/reductases (SDRs) family^32^, which participates in steroid and retinol metabolism^33^. RDH12, a NADPH-dependent all-trans retinol dehydrogenase, is the key enzyme in the metabolism of retinoids^34^. Two oxidation products of retinoids, 9-cis-retinoic acid and all-trans retinoic acid, function to stimulate insulin secretion^35^. In adipocytes, retinoic acid induces the expression of the insulin signaling gene *PDK-1* and that of the glucose transporter GLUT4. Activating retinoic acid induces the expression of genes involved in lipid and glucose metabolism, thereby improving insulin action^36^. Thus, hypermethylation of the CpG 68167386 located upstream of the promoter region of *RDH12* may inhibit its transcriptional activity and reduce RDH12 levels in peripheral blood. Subsequently, the retinoic acid metabolic pathway would be inhibited, affecting insulin secretion, and reducing its effectiveness.

The DNAJB6 (DnaJ homolog, subfamily B, member 6) protein is a member of the heat shock protein 40 (HSP40) family^37^ and acts as a molecular chaperone for various cellular processes. While observing insulin resistant and diabetic patients, Kurucz et al.^38^ found that *HSP* expression was significantly changed without diabetes, and that the mRNA level of HSP72-inducible subtypes was significantly reduced in patients with type 2 diabetes. Additionally, the expression of *HSP70* in the skeletal muscle of patients with type 2 diabetes is reduced and has been shown to correlate with the degree of insulin resistance^39^. These HSP molecular chaperones are related to diabetes^40^. However, the exact association between DNAJB6 and type 2 diabetes needs further study. In this study, hypermethylation of CpG sites 157130156, located in the promoter region of DNAJB6, was observed in the GDM group. This might result in increased DNAJB6 levels via the up-regulation of *DNABJ6* transcription, thereby reducing the risk of GDM (OR = 0.361; 95% CI 0.135–0.966).

*NFATC4* codes the nuclear factor of activated T cells 4 (NFATC4), which is a member of the transcription factor family under the control of calcineurin (a Ca^2+^-dependent phosphatase)^41^. In adipose tissue, NFATC4 has been shown to promote the secretion of inflammatory factors^42^, and to act as a transcriptional repressor in regulating adiponectin gene expression, suggesting that adiponectin expression is down-regulated in obesity and type 2 diabetes^43^. In this study, hypermethylation of the CpG site 24837915 located in the promoter region of *NFATC4*, was associated with the presence of GDM (OR = 5.232; 95% CI 1.659–16.506).

During pregnancy, early anabolism increases and mild insulin resistance occurs^44^. When insulin secretion fails to balance insulin resistance, impaired glucose tolerance develops, which might subsequently lead to GDM^45^. Therefore, impaired secretion by β cells is also a key factor in GDM pathogenesis. Here, we explored the pathogenesis of GDM from an epigenetic perspective and identified 13 CpG sites that had methylation levels showing associations with GDM pathogenesis. Furthermore, conditional logistic regression analysis showed that the methylation status of four CpG sites located in the promoter regions of four genes was associated with GDM pathogenesis. These CpG sites are located in genes that could contribute to the development of GDM. Of these four CpG sites, hypermethylation of CpG site 24837915 and CpG site 68167324 was shown to be associated with GDM, whereas that of CpG site 89438648 and CpG site 157130156 could indicate reduced risk of GDM. Thus, the methylation status of these genes may function as predictors of GDM. No publications reporting on the relationship between methylation of these four CpG sites and GDM have been found, so our suggestion of such a relationship is based on the known modes of action of the genes concerned.

However, the study also had some limitations. First, the selection of our target CpG sites was based on published literature, and we did not screen for differential CpG sites in the same population in this study, so there may be other CpG sites related to the pathogenesis of GDM that have not been verified. Second, probably due to the large total number of detected CpG sites in the background, as well as the limited significant different sites, no significant differences were found anymore after the FDR correction was used for the 337 tests. Third, the blood samples were centrifuged to separate serum and blood cells within 24 h after collection, but the cell type composition was not further separated. Which prevented us from considering DNA methylation on cell type composition or the adjustment for cell proportions. Finally, since that our sample size was relatively limited, these findings need to be further verified using a larger and broader population.

## Conclusions

In summary, by determining the DNA methylation of target CpG sites in the peripheral blood of women in early pregnancy, we found that the methylation levels of 13 CpG sites were related to GDM by quantitative analysis. After adjusting for possible confounding factors by conditional logistic regression, four CpG sites showed independent effects on GDM. These findings indicate that the methylation status of these CpG sites in the peripheral blood of pregnant women during the first trimester might be associated with was related to the pathogenesis of GDM. But the exact relationship still needs further research and verification.

## Supplementary Information

Supplementary Information 1.

Supplementary Information 2.

## Data Availability

Raw data of patient characteristics and the data of target fragment DNA methylation sequencing generated during the current study is available from the corresponding author upon reasonable request.

## Abbreviations

GDM: Gestational diabetes
ROC: Receiver operating characteristic curves
OGTT: Oral glucose tolerance test
