# Supplementary material for: Study on the relationship between DNA methylation of target CpG sites in peripheral blood and gestational diabetes during early pregnancy
Source: Sci Rep. 2021 Oct 14;11:20455. doi: 10.1038/s41598-021-99836-2 (PMC8516930; doi:10.1038/s41598-021-99836-2)
Supplement: Supplementary file 2 — Supplementary Information 2. [file 41598_2021_99836_MOESM2_ESM.docx]

**Supplementary materials**

[Supplementary Table S1 2](#_Toc25314282)

[Supplementary Table S2 4](#_Toc25314283)

[Supplementary Table S3. 6](#_Toc25314284)

[Supplementary Table S4. 7](#_Toc25314285)

[Supplementary Table S5 11](#_Toc25314286)

[Supplementary Figure S1 12](#_Toc25314286)

[Reference 14](#_Toc25314288)

| Supplementary Table S1: The basic information of the sequenced fragments | | | | | | | |
| --- | --- | --- | --- | --- | --- | --- | --- |
| Target | Chr | Gene | **Gene full name** | **TSS** | **TES** | **Start** | **End** |
| ARHGAP40_16 | 20 | ARHGAP40 | The RHO Guanosine triphosphatase activating protein 40 | 37230576 | 37279295 | 37274153 | 37274356 |
| C5orf34_15 | 5 | C5orf34 | Chromosome 5 open reading frame 34 | 43515273 | 43486802 | 43487447 | 43487684 |
| CCDC124_14 | 19 | CCDC124 | Coiled-coil domain containing 124 | 18045904 | 18054794 | 18054774 | 18054574 |
| COPS8_11 | 2 | COPS8 | Constitutive photomorphogenic homolog subunit 8 | 237994083 | 238007489 | 237992528 | 237992710 |
| DDR1_05 | 6 | DDR1 | Discoid in domain receptor tyrosine kinase 1 | 30852756 | 30867933 | 30852639 | 30852815 |
| DNAJB6_09 | 7 | DNAJB6 | DNAJ homolog, subfamily B, member 6 | 157129710 | 157210133 | 157130190 | 157130031 |
| EIF2AK2_28 | 2 | EIF2AK2 | Eukaryotic translation initiation factor 2α kinase 2. | 37374965 | 37332283 | 37375038 | 37374822 |
| GCK_18 | 7 | GCK | Glucokinase | 44185743 | 44182811 | 44185089 | 44185314 |
| HAAO_13 | 2 | HAAO | 3-hydroxyanthranilate3,4-dioxygenase | 43019751 | 42994228 | 43013714 | 43013885 |
| HAPLN3_02 | 15 | HAPLN3 | Hyaluronan and proteoglycan link protein 3 | 89438857 | 89420515 | 89438585 | 89438810 |
| HHLA3_03 | 1 | HHLA3 | HERV-H LTR-associating 3 | 70820492 | 70833705 | 70820116 | 70820310 |
| IFNGR2_27 | 21 | IFNGR2 | The interferon gamma receptor 2 | 34775201 | 34809828 | 34775191 | 34775397 |
| IL10_25 | 1 | IL10 | Interleukin 10 | 206945839 | 206940946 | 206945211 | 206945446 |
| IL6_21 | 7 | IL6 | Interleukin 6 | 22766760 | 22771621 | 22765200 | 22765453 |
| IL6_22 | 7 | IL6 | Interleukin 6 | 22766760 | 22771621 | 22767361 | 22767112 |
| IL7_10 | 8 | IL7 | Interleukin 7 | 79717758 | 79645006 | 79717646 | 79717816 |
| MDM2_08 | 12 | MDM2 | MDM2 oncogene, E3 ubiquitin protein ligase | 69201951 | 69239324 | 69202015 | 69202223 |
| NAGA_30 | 22 | NAGA | a-N-Acetylgalactosaminidase | 42466846 | 42454337 | 42466437 | 42466229 |
| NDUFC1_01 | 4 | NDUFC1 | NADH dehydrogenase (ubiquinone) 1, subcomplex unknown | 140222364 | 140211070 | 140216838 | 140217048 |
| NFATC4_17 | 14 | NFATC4 | Nuclear factor of activated T cells 4 | 24838203 | 24848810 | 24837955 | 24837799 |
| PIK3R5_12 | 17 | PIK3R5 | Phosphoinositide-3-kinase, regulatory subunit 5 | 8815834 | 8782232 | 8792251 | 8792063 |
| PPARG_20 | 3 | PPARG | peroxisome proliferator‐activated receptor gamma | 12329348 | 12475855 | 12329707 | 12329966 |
| RDH12_23 | 14 | RDH12 | retinol dehydrogenase 12 | 68168602 | 68201168 | 68166518 | 68166775 |
| RDH12_24 | 14 | RDH12 | retinol dehydrogenase 12 | 68168602 | 68201168 | 68167191 | 68167424 |
| RHOG_04 | 11 | RHOG | ras homolog family member G | 3862213 | 3848207 | 3863137 | 3862904 |
| SEPT11_07 | 4 | SEPT11 | Septin 11 | 77870866 | 77959768 | 77871462 | 77871255 |
| STAT1_26 | 2 | STAT1 | The signal transducer and activator of transcription 1 | 191878976 | 191833761 | 191879029 | 191879241 |
| YAP1_29 | 11 | YAP1 | Yes-associated protein | 101981191 | 102104154 | 101980889 | 101981108 |
| ZAR1_06 | 4 | ZAR1 | Zygote arrest 1 | 48492268 | 48496406 | 48492464 | 48492222 |
| Notes: Target: Target fragment of DNA sequencing; Chr: Chromosome; Gene: The name of the gene in which the target fragment is located; TSS: mRNA transcription initiation site; TES: mRNA transcription termination sites; Start: Target fragment initiation site; End: Target fragment termination site; Length: Target fragment sequencing length; Distance to TSS: The distance of the target fragment from the TSS. The minus sign indicates that the target is upstream of the transcription start site. | | | | | | | |

| Supplementary Table S2: Primer information of the target fragment | | | | |
| --- | --- | --- | --- | --- |
| Target | Length | Distance to TSS | Primer F | Primer R |
| ARHGAP40_16 | 204 | 43577 | AAAAGGGGAATTTAGAATYGATTTTAGT | ACACCTAAAACAAACTCTACTCTAAAAACC |
| C5orf34_15 | 238 | 27826 | GGTTTGGTGGTAAGGAGGAAATG | ATTCTATACATTCTCACCCAATTCATCT |
| CCDC124_14 | 201 | 8870 | GTTATTTTTATTGGGTATTTAGTTTTGAGGT | TCTACACRCCCTTAAACCAAATCAA |
| COPS8_11 | 183 | -1555 | GAGGTTAGTAAGATTTAAAGTGAGTATAAGGTA | ACCATAACTCATACAATATAATTTCTTCATAC |
| DDR1_05 | 177 | -117 | TTTYGATGTTTTGGTATATYGTTTGAA | AACTAACTCAATAAAACTACACAAAAACACC |
| DNAJB6_09 | 160 | 480 | GGTTTYGTYGAGTTTTAGTYGAGTTTTAGT | ATCTAAAACCRAAACCRAAACTAAAATCT |
| EIF2AK2_28 | 217 | -73 | TAAATTGTATTGGGAAATTTAGATTAAATATGTT | ACAACCTACCTCCTATCATATAAAAATCCTA |
| GCK_18 | 226 | 654 | GGTTTATTTGGGGTGTAGTTTGT | CAAACAAATCTACAACATCCTAAACA |
| HAAO_13 | 172 | 6037 | TTATGTGTTTATGGAAATAGGTTTGGAGA | CACCTACCACCTATACTCCACCTACC |
| HAPLN3_02 | 226 | 272 | GATTTTGTTYGGGAGGTGTGG | AATTTTTCTACTACCRCTTCCTTTCTACC |
| HHLA3_03 | 195 | -376 | GAATYGGGAGGTTTATYGTTGG | AATTCTAACTTCCCCCAACRAAC |
| IFNGR2_27 | 207 | -10 | TTTAAAATGGGGTTGATTGGAG | CTCTCCTCTCRAATACTTAATCRCACAATTC |
| IL10_25 | 236 | 628 | TTTAAATTATTGGTTTTTTAGGTTTGGAA | ATTAAACTAAACCAAATAATACAATAAATATACAAA |
| IL6_21 | 254 | -1560 | TTATGTAGGAAAGAGAATTTGGTTTAGG | TAAAACAATAACCTCTATTAAACATTTACTCAA |
| IL6_22 | 250 | 601 | TTAGAATTTAGTAAAGATTTTTTAATGTAGGTAGT | TACTACCTTCCCTACCCCAATACC |
| IL7_10 | 171 | 112 | GATTGGGAGTTAGAATTATGAGTTGTTAATG | TAACCCTCTTAATCATTCTTCACTTCCTT |
| MDM2_08 | 209 | 64 | TGTGTYGGAAAGATGGAGTAAGAAGT | AAAACTCCCCAATTTCCTTCAC |
| NAGA_30 | 209 | 409 | TTGAGGGYGGTYGAGTTAGTTAGGTAGT | ACACTCCTTATAACACTCACCCCTACC |
| NDUFC1_01 | 211 | 5526 | GAGGGGTAATTTGAGGGTYGAGT | AAACTCTACTACAAAACCCTCRAATCC |
| NFATC4_17 | 157 | -248 | TTTGGYGGYGTTTGGTGTTGTT | AACTAAAAACCCRAACACCACTTTC |
| PIK3R5_12 | 189 | 23583 | GGGGTTTAGAYGTTTAGATAGTGTTGG | AAACAAAAAATCTCTCTCRACACAATAC |
| PPARG_20 | 260 | 359 | GGTAGGGTTATGGTTYGGTAGGATT | TCCCCRTATCCCCRACTCC |
| RDH12_23 | 258 | -2084 | TTGGAATTTTATTTAGATAATTTGGAAGAT | AAACRATTCTCCTACCTCCATCTCC |
| RDH12_24 | 234 | -1411 | GGGAGGTYGAGGTGGGTAGA | AATCTTACTATATCACCCAAACTAAAATACAATAC |
| RHOG_04 | 234 | -924 | ATTTGAAAAGTTTTTGGAAGATAGG | CCCAAACCTAAAATCCAACTTAAAC |
| SEPT11_07 | 208 | 596 | GGGGAGATTGGAGGAAAGTT | CCCAAAAACTCAATTACRTAATACAACAATA |
| STAT1_26 | 213 | -53 | GTTTYGTTTAYGYGTTGGGGTATT | ATCTATCCTCTACCTAAATTCTCRACRATAA |
| YAP1_29 | 220 | -302 | GTTYGYGGGGTAGAATAYGGGGTA | CTCCTCTCRACTCTTCCTTCCTCTA |
| ZAR1_06 | 243 | 196 | GGAGGAGGYGGGAAGGTAGTT | CCCCRCCRAAAACCATCC |
| Notes: Length: Target fragment sequencing length; Distance to TSS: The distance of the target fragment from the TSS. The minus sign indicates that the target is upstream of the transcription start site; Primer F: Target fragment forward primer sequence; Primer R: Target fragment reverse primer sequence. | | | | |

| Supplementary Table S3: Source of candidate CpG sites | | | |
| --- | --- | --- | --- |
| References | Method | Gene Symbol | Number of CpG islands or cg sites |
| Enquobahrie 2015 ^1^ | Epigenome-Wide Methylation Profiling | HAPLN3 | cg 08348496 |
|  |  | HHLA3 | cg 09735905 |
|  |  | RHOG | cg 17732521 |
|  |  | MDM2 | cg 12504957 |
|  |  | DNAJB6 | cg 13456653 |
|  |  | IL7 | cg 23512958 |
|  |  | YAP1 | cg 01442799 |
|  |  | NAGA | cg 27187881 |
|  |  | NDUFC1 | cg 15010390 |
|  |  | SEPT11 | cg 00899086 |
|  |  | DDR1 | cg 11977634 |
|  |  | ZAR1 | cg 18342279 |
|  |  |  |  |
| Li 2019 ^6^ | Gene Ontology (GO) and Kyoto Encyclopedia of Genes and Genomes (KEGG) pathway enrichment analysis | STAT1 | cg 07052015 |
|  |  | IFNGR2 | cg 23508786 |
|  |  | EIF2AK2 | cg 06969118 |
|  |  |  |  |
| Wu 2018^2^ | Genome-Wide DNA Methylation Profiling (450K) | COPS8 | cg 16995742 |
|  |  | PIK3R5 | cg 02823329 |
|  |  | HAAO | cg 17283620 |
|  |  | CDCC124 | cg 14060113 |
|  |  | C5orf34 | cg 09101062 |
|  |  | RDH12 | 2 |
|  |  |  |  |
| Kang 2018 ^5^ | MethylCode^TM^ Bisulfite Conversion Kit | IL10 | cg206771801- cg206772098 |
|  |  |  |  |
| Laura 2014 ^3^ |  | GCK | 1 |
|  |  |  |  |
| Naidoo 2018 ^4^ |  | PPARG | 1 |
|  |  | IL6 | 2 |
|  |  |  |  |
| Bioinformatics Analysis |  | ARHGAP40 | cg 03044376 |
|  |  | NFATC4 | cg 06111454 |

|  |  | | Supplementary Table S4: Univariate analysis of 337 CpG sites | | | | | | | | | |
| --- | --- | --- | --- | --- | --- | --- | --- | --- | --- | --- | --- | --- |
| Gene Symbol | | CpG site | | Chr | Missing values | P-value | | Gene Symbol | CpG site | Chr | Missing values | P-value |
| ARHGAP40 | | 37274183 | | 20 | 0 | 0.388 | EIF2AK2_28 | | 37374961* | 2 | 0 | 0.222 |
|  | | 34274257* | | 20 | 0 | 0.015 |  | | 37374898 | 2 | 0 | 0.231 |
| C5orf34 | | 43487508 | | 5 | 0 | 0.012 | DDR1_05 | | 30852669 | 6 | 1 | 0.469 |
|  | | 43487597* | | 5 | 0 | 0.612 |  | | 30852675 | 6 | 1 | 0.561 |
| CCDC124 | | 18054733 | | 19 | 0 | 0.850 |  | | 30852699 | 6 | 1 | 0.988 |
|  | | 18054731 | | 19 | 0 | 0.673 |  | | 30852761 | 6 | 1 | 0.278 |
|  | | 18054728 | | 19 | 0 | 0.524 |  | | 30852777 | 6 | 1 | 0.945 |
|  | | 18054699 | | 19 | 0 | 0.558 | GCK_18 | | 44185115* | 7 | 0 | 0.505 |
|  | | 18054680 | | 19 | 0 | 0.163 |  | | 44185127* | 7 | 0 | 0.797 |
|  | | 18054672 | | 19 | 0 | 0.130 |  | | 44185141 | 7 | 0 | 0.407 |
|  | | 18054644 | | 19 | 0 | 0.708 |  | | 44185148 | 7 | 0 | 0.160 |
|  | | 18054637 | | 19 | 0 | 0.248 |  | | 44185154 | 7 | 0 | 0.096 |
|  | | 18054634 | | 19 | 0 | 0.844 |  | | 44185159 | 7 | 0 | 0.777 |
|  | | 18054622 | | 19 | 0 | 0.312 |  | | 44185166 | 7 | 0 | 0.555 |
|  | | 18054617 | | 19 | 0 | 0.380 |  | | 44185168 | 7 | 0 | 0.863 |
|  | | 18054603 | | 19 | 0 | 0.822 |  | | 44185174 | 7 | 0 | 0.698 |
| COPS8_11 | | 237992563 | | 2 | 0 | 0.305 |  | | 44185184 | 7 | 0 | 0.916 |
|  | | 237992612 | | 2 | 0 | 0.242 |  | | 44185188 | 7 | 0 | 0.950 |
|  | | 237992645 | | 2 | 0 | 0.138 |  | | 44185197 | 7 | 0 | 0.199 |
| DNAJB6_09 | | 157130160 | | 7 | 1 | 0.992 |  | | 44185200 | 7 | 0 | 0.443 |
|  | | 157130156 | | 7 | 1 | 0.013 |  | | 44185212* | 7 | 0 | 0.357 |
|  | | 157130145 | | 7 | 1 | 0.287 |  | | 44185217 | 7 | 0 | 0.673 |
|  | | 157130128 | | 7 | 1 | 0.837 |  | | 44185219 | 7 | 0 | 0.432 |
|  | | 157130125 | | 7 | 1 | 0.415 |  | | 44185221 | 7 | 0 | 0.814 |
|  | | 157130122 | | 7 | 1 | 0.104 |  | | 44185229* | 7 | 0 | 0.132 |
|  | | 157130120 | | 7 | 1 | 0.067 |  | | 44185235* | 7 | 0 | 0.188 |
|  | | 157130108 | | 7 | 1 | 0.844 |  | | 44185241 | 7 | 0 | 0.056 |
|  | | 157130106 | | 7 | 1 | 0.345 |  | | 44185243 | 7 | 0 | 0.240 |
|  | | 157130101 | | 7 | 1 | 0.594 |  | | 44185246* | 7 | 0 | 0.393 |
|  | | 157130092 | | 7 | 1 | 0.129 |  | | 44185250 | 7 | 0 | 0.357 |
|  | | 157130089 | | 7 | 1 | 0.252 |  | | 44185256 | 7 | 0 | 0.270 |
|  | | 157130085 | | 7 | 1 | 0.018 |  | | 44185262* | 7 | 0 | 0.364 |
|  | | 157130076 | | 7 | 1 | 0.575 |  | | 44185269* | 7 | 0 | 0.517 |
|  | | 157130074 | | 7 | 1 | 0.966 |  | | 44185276* | 7 | 0 | 0.256 |
|  | | 157130070 | | 7 | 1 | 0.766 |  | | 44185287 | 7 | 0 | 0.916 |
|  | | 157130066 | | 7 | 1 | 0.578 | HHLA3_03 | | 70820138 | 1 | 0 | 0.539 |
| HAAO_13 | | 43013772 | | 2 | 0 | 0.601 |  | | 70820141* | 1 | 0 | 0.982 |
|  | | 43013785 | | 2 | 0 | 0.289 |  | | 70820150* | 1 | 0 | 0.337 |
|  | | 43013841 | | 2 | 0 | 0.759 |  | | 70820155* | 1 | 0 | 0.946 |
| HAPLN3_02 | | 89438611* | | 15 | 1 | 0.471 |  | | 70820158* | 1 | 0 | 0.720 |
|  | | 89438627 | | 15 | 1 | 0.996 |  | | 70820168* | 1 | 0 | 0.074 |
|  | | 89438643 | | 15 | 1 | 0.581 |  | | 70820177* | 1 | 0 | 0.482 |
|  | | 89438648 | | 15 | 1 | 0.048 |  | | 70820197* | 1 | 0 | 0.771 |
|  | | 89438671 | | 15 | 1 | 0.145 |  | | 70820223* | 1 | 0 | 0.484 |
|  | | 89438685 | | 15 | 1 | 0.085 |  | | 70820238* | 1 | 0 | 0.109 |
|  | | 89438690 | | 15 | 1 | 0.708 |  | | 70820247* | 1 | 0 | 0.499 |
|  | | 89438708* | | 15 | 1 | 0.889 |  | | 70820258* | 1 | 0 | 0.237 |
|  | | 89438711* | | 15 | 1 | 0.791 |  | | 70820265* | 1 | 0 | 0.907 |
|  | | 89438724 | | 15 | 1 | 0.460 |  | | 70820275* | 1 | 0 | 0.762 |
|  | | 89438731* | | 15 | 1 | 0.862 |  | | 70820283* | 1 | 0 | 0.316 |
|  | | 89438733 | | 15 | 1 | 0.355 |  | | 70820285* | 1 | 0 | 0.909 |
|  | | 89438743 | | 15 | 1 | 0.401 |  | | 70820287 | 1 | 0 | 0.878 |
|  | | 89438767 | | 15 | 1 | 0.101 | IL10_25 | | 206945241* | 1 | 2 | 0.427 |
| IFNGR2_27 | | 34775214 | | 21 | 8 | 0.958 |  | | 206945301* | 1 | 2 | 0.376 |
|  | | 34775231 | | 21 | 8 | 0.740 |  | | 206945346 | 1 | 2 | 0.316 |
|  | | 34775237 | | 21 | 8 | 0.361 |  | | 206945376 | 1 | 2 | 0.912 |
|  | | 34775266 | | 21 | 8 | 0.956 |  | | 206945386 | 1 | 2 | 0.330 |
|  | | 34775291 | | 21 | 8 | 0.222 | IL7_10 | | 79717683 | 8 | 0 | 0.958 |
|  | | 34775346 | | 21 | 8 | 0.490 |  | | 79717705* | 8 | 0 | 0.836 |
|  | | 34775358 | | 21 | 8 | 0.012 |  | | 79717726 | 8 | 0 | 0.962 |
|  | | 34775366 | | 21 | 8 | 0.191 |  | | 79717730* | 8 | 0 | 0.310 |
| IL6_21 | | 22767323* | | 7 | 1 | 0.889 |  | | 79717744* | 8 | 0 | 0.999 |
|  | | 22767315* | | 7 | 1 | 0.406 |  | | 79717750 | 8 | 0 | 0.638 |
|  | | 22767298* | | 7 | 1 | 0.183 |  | | 79717752 | 8 | 0 | 0.412 |
|  | | 22767292 | | 7 | 1 | 0.454 |  | | 79717765 | 8 | 0 | 0.715 |
|  | | 22767287 | | 7 | 1 | 0.408 |  | | 79717776 | 8 | 0 | 0.645 |
|  | | 22767269 | | 7 | 1 | 0.615 | MDM2_08 | | 69202041* | 12 | 0 | 0.143 |
|  | | 22767230* | | 7 | 1 | 0.921 |  | | 69202047* | 12 | 0 | 0.060 |
|  | | 22767227* | | 7 | 1 | 0.699 |  | | 69202054 | 12 | 0 | 0.484 |
|  | | 22767216* | | 7 | 1 | 0.684 |  | | 69202058* | 12 | 0 | 0.365 |
|  | | 22767198* | | 7 | 1 | 0.668 |  | | 69202060 | 12 | 0 | 0.618 |
|  | | 22767164* | | 7 | 1 | 0.283 |  | | 69202073 | 12 | 0 | 0.741 |
| NAGA_30 | | 42466409 | | 22 | 1 | 0.631 |  | | 69202090* | 12 | 0 | 0.834 |
|  | | 42466387 | | 22 | 1 | 0.133 |  | | 69202105 | 12 | 0 | 0.499 |
|  | | 42466346 | | 22 | 1 | 0.575 |  | | 69202115 | 12 | 0 | 0.426 |
|  | | 42466321* | | 22 | 1 | 0.008 |  | | 69202125 | 12 | 0 | 0.897 |
|  | | 42466303 | | 22 | 1 | 0.145 |  | | 69202129 | 12 | 0 | 0.581 |
|  | | 42466275 | | 22 | 1 | 0.209 |  | | 69202133* | 12 | 0 | 0.918 |
| NDUFC1_01 | | 140216861 | | 4 | 0 | 0.916 |  | | 69202150* | 12 | 0 | 0.301 |
|  | | 140216870* | | 4 | 0 | 0.279 |  | | 69202169 | 12 | 0 | 0.604 |
|  | | 140216885 | | 4 | 0 | 0.719 |  | | 69202181 | 12 | 0 | 0.316 |
|  | | 140216889* | | 4 | 0 | 0.258 |  | | 69202184* | 12 | 0 | 0.346 |
|  | | 140216893* | | 4 | 0 | 0.902 |  | | 69202192* | 12 | 0 | 0.844 |
|  | | 140216904 | | 4 | 0 | 0.740 |  | | 69202194* | 12 | 0 | 0.621 |
|  | | 140216918 | | 4 | 0 | 0.943 |  | | 69202196 | 12 | 0 | 0.989 |
|  | | 140216930 | | 4 | 0 | 0.101 |  | | 69202201 | 12 | 0 | 0.350 |
|  | | 140216940 | | 4 | 0 | 0.807 | NFATC4_17 | | 24837928* | 14 | 0 | 0.577 |
|  | | 140216944 | | 4 | 0 | 0.635 |  | | 24837919 | 14 | 0 | 0.502 |
|  | | 140216947 | | 4 | 0 | 0.663 |  | | 24837915* | 14 | 0 | 0.018 |
|  | | 140216957 | | 4 | 0 | 0.777 |  | | 24837893* | 14 | 0 | 0.055 |
|  | | 140216978* | | 4 | 0 | 0.784 |  | | 24837884* | 14 | 0 | 0.163 |
|  | | 140216998 | | 4 | 0 | 0.601 |  | | 24837877* | 14 | 0 | 0.864 |
|  | | 140217008 | | 4 | 0 | 0.266 |  | | 24837874* | 14 | 0 | 0.102 |
|  | | 140217012 | | 4 | 0 | 0.931 |  | | 24837837* | 14 | 0 | 0.098 |
|  | | 140217020 | | 4 | 0 | 0.401 | PIK3R5_12 | | 8792093 | 17 | 0 | 0.825 |
| RDH12 | | 68166584* | | 14 | 0 | 0.383 | PPARG_20 | | 12329732* | 3 | 0 | 0.732 |
|  | | 68166586* | | 14 | 0 | 0.895 |  | | 12329740* | 3 | 0 | 0.658 |
|  | | 68166596 | | 14 | 0 | 0.848 |  | | 12329745* | 3 | 0 | 0.523 |
|  | | 68166624* | | 14 | 0 | 0.981 |  | | 12329747* | 3 | 0 | 0.974 |
|  | | 68166633 | | 14 | 0 | 0.830 |  | | 12329751 | 3 | 0 | 0.342 |
|  | | 68166654* | | 14 | 0 | 0.301 |  | | 12329755 | 3 | 0 | 0.698 |
|  | | 68166685* | | 14 | 0 | 0.970 |  | | 12329762 | 3 | 0 | 0.372 |
|  | | 68166717* | | 14 | 0 | 0.748 |  | | 12329770 | 3 | 0 | 0.640 |
|  | | 68166727* | | 14 | 0 | 0.758 |  | | 12329800 | 3 | 0 | 0.673 |
|  | | 68167214* | | 14 | 0 | 0.569 |  | | 12329812 | 3 | 0 | 0.536 |
|  | | 68167228 | | 14 | 0 | 0.924 |  | | 12329814 | 3 | 0 | 0.481 |
|  | | 68167248 | | 14 | 0 | 0.601 |  | | 12329821* | 3 | 0 | 0.892 |
|  | | 68167259 | | 14 | 0 | 0.157 |  | | 12329826 | 3 | 0 | 0.730 |
|  | | 68167288 | | 14 | 0 | 0.119 |  | | 12329828 | 3 | 0 | 0.770 |
|  | | 68167292 | | 14 | 0 | 0.770 |  | | 12329839 | 3 | 0 | 0.502 |
|  | | 68167300* | | 14 | 0 | 0.605 |  | | 12329846* | 3 | 0 | 0.786 |
|  | | 68167304 | | 14 | 0 | 0.472 |  | | 12329848* | 3 | 0 | 0.502 |
|  | | 68167324* | | 14 | 0 | 0.019 |  | | 12329862 | 3 | 0 | 0.305 |
|  | | 68167348* | | 14 | 0 | 0.352 |  | | 12329864* | 3 | 0 | 0.364 |
|  | | 68167363 | | 14 | 0 | 0.642 |  | | 12329868* | 3 | 0 | 0.630 |
|  | | 68167386 | | 14 | 0 | 0.042 |  | | 12329874* | 3 | 0 | 0.692 |
|  | | 68167388 | | 14 | 0 | 0.818 |  | | 12329879* | 3 | 0 | 0.330 |
| RHOG_04 | | 3863098* | | 11 | 0 | 0.913 |  | | 12329887 | 3 | 0 | 0.792 |
|  | | 3863057* | | 11 | 0 | 0.771 |  | | 12329889* | 3 | 0 | 0.240 |
|  | | 3863048 | | 11 | 0 | 0.737 |  | | 12329894* | 3 | 0 | 0.716 |
|  | | 3863026* | | 11 | 0 | 0.383 |  | | 12329899 | 3 | 0 | 0.054 |
|  | | 3862996 | | 11 | 0 | 0.946 |  | | 12329904 | 3 | 0 | 0.822 |
|  | | 3862992 | | 11 | 0 | 0.236 |  | | 12329926* | 3 | 0 | 0.291 |
|  | | 3862977* | | 11 | 0 | 0.789 |  | | 12329934 | 3 | 0 | 0.981 |
|  | | 3862962 | | 11 | 0 | 0.352 |  | | 12329937* | 3 | 0 | 0.574 |
|  | | 3862950* | | 11 | 0 | 0.423 |  | | 12329945 | 3 | 0 | 0.870 |
|  | | 3862942 | | 11 | 0 | 0.181 |  | | 12329947* | 3 | 0 | 0.571 |
|  | | 3862940 | | 11 | 0 | 0.562 | SEPT11_07 | | 77871441 | 4 | 0 | 0.973 |
|  | | 3862935 | | 11 | 0 | 0.927 |  | | 77871435* | 4 | 0 | 0.945 |
|  | | 3862932 | | 11 | 0 | 0.852 |  | | 77871432* | 4 | 0 | 0.628 |
| STAT1_26 | | 191879055* | | 2 | 0 | 0.264 |  | | 77871415 | 4 | 0 | 0.05 |
|  | | 191879058 | | 2 | 0 | 0.309 |  | | 77871401 | 4 | 0 | 0.752 |
|  | | 191879065* | | 2 | 0 | 0.925 |  | | 77871399 | 4 | 0 | 0.496 |
|  | | 191879068 | | 2 | 0 | 0.676 |  | | 77871385 | 4 | 0 | 0.822 |
|  | | 191879075 | | 2 | 0 | 0.870 |  | | 77871374 | 4 | 0 | 0.487 |
|  | | 191879078* | | 2 | 0 | 0.625 |  | | 77871371 | 4 | 0 | 0.601 |
|  | | 191879104 | | 2 | 0 | 0.037 |  | | 77871369* | 4 | 0 | 0.781 |
|  | | 191879114 | | 2 | 0 | 0.205 |  | | 77871319 | 4 | 0 | 0.211 |
|  | | 191879127 | | 2 | 0 | 0.588 |  | | 77871300 | 4 | 0 | 0.708 |
|  | | 191879152 | | 2 | 0 | 0.837 |  | | 77871298 | 4 | 0 | 0.195 |
|  | | 191879164 | | 2 | 0 | 0.161 |  | | 77871295 | 4 | 0 | 0.781 |
| ZAR1_06 | | 48492438* | | 4 | 0 | 0.977 |  | | 77871290* | 4 | 0 | 0.914 |
|  | | 48492436 | | 4 | 0 | 0.992 | YAP1_29 | | 101980913* | 11 | 0 | 0.145 |
|  | | 48492420 | | 4 | 0 | 0.604 |  | | 101980918 | 11 | 0 | 0.837 |
|  | | 48492416* | | 4 | 0 | 0.558 |  | | 101980930* | 11 | 0 | 0.203 |
|  | | 48492413 | | 4 | 0 | 0.874 |  | | 101980938 | 11 | 0 | 0.870 |
|  | | 48492411* | | 4 | 0 | 0.651 |  | | 101980940 | 11 | 0 | 0.412 |
|  | | 48492392* | | 4 | 0 | 0.306 |  | | 101980944* | 11 | 0 | 0.176 |
|  | | 48492390 | | 4 | 0 | 0.135 |  | | 101980951* | 11 | 0 | 0.964 |
|  | | 48492376* | | 4 | 0 | 0.399 |  | | 101980956* | 11 | 0 | 0.458 |
|  | | 48492371* | | 4 | 0 | 0.347 |  | | 101980966 | 11 | 0 | 0.807 |
|  | | 48492356 | | 4 | 0 | 0.254 |  | | 101980970* | 11 | 0 | 0.596 |
|  | | 48492353 | | 4 | 0 | 0.159 |  | | 101980977* | 11 | 0 | 0.970 |
|  | | 48492345* | | 4 | 0 | 0.860 |  | | 101980981* | 11 | 0 | 0.831 |
|  | | 48492339* | | 4 | 0 | 0.841 |  | | 101980985* | 11 | 0 | 0.157 |
|  | | 48492327* | | 4 | 0 | 0.617 |  | | 101980994* | 11 | 0 | 0.962 |
|  | | 48492314* | | 4 | 0 | 0.121 |  | | 101980997* | 11 | 0 | 0.475 |
|  | | 48492305 | | 4 | 0 | 0.252 |  | | 101980999* | 11 | 0 | 0.001 |
|  | | 48492294* | | 4 | 0 | 0.543 |  | | 101981004* | 11 | 0 | 0.191 |
|  | | 48492290 | | 4 | 0 | 0.321 |  | | 101981035 | 11 | 0 | 0.840 |
|  | | 48492287 | | 4 | 4 | 0.848 |  | | 101981050 | 11 | 0 | 0.107 |
|  | | 48492284* | | 4 | 0 | 0.252 |  | | 101981056* | 11 | 0 | 0.519 |
|  | | 48492269* | | 4 | 0 | 0.604 |  | | 101981060* | 11 | 0 | 0.028 |
|  | | 48492260 | | 4 | 0 | 0.807 |  | | 101981065 | 11 | 0 | 0.912 |
|  | | 48492256* | | 4 | 0 | 0.318 |  | | 101981067 | 11 | 0 | 0.135 |
|  | | 48492252* | | 4 | 0 | 0.747 |  | | 101981072 | 11 | 0 | 0.077 |
|  | | 48492248* | | 4 | 0 | 0.468 |  | | 101981076 | 11 | 0 | 0.120 |
|  | | 48492242 | | 4 | 0 | 0.524 |  | | 101981080* | 11 | 0 | 0.593 |
|  | |  | |  |  |  |  | | 101981082 | 11 | 0 | 0.908 |
| *: Normal distribution; Paired-samples T test were used to compare normally distributed continuous data, whereas Wilcoxon signed rank test were used to analyze non-normally distributed continuous data | | | | | | | | | | | | |

| Supplementary Table S5: A brief introduction to the function of the genes where those differential CpG sites are located | |
| --- | --- |
| Gene Symbol | Function summary |
| ARHGAP40 | It involved in cell cycle progression, cytoskeleton reorganization, cell polarity, migration, and invasion. |
| C5orf34 | It involved in gene regulation and cell proliferation. |
| RDH12 | It participates in the metabolism of steroids and retinol. |
| YAP1 | It interacts with DNA-binding proteins to promote proliferation and inhibit cell apoptosis. |
| NAGA | It hydrolyzes the α-N-acetylglucosamine bond in glycoconjugates. |
| HAPLN3 | Aggregation of proteoglycan and hyaluronic acid, cell adhesion. |
| DNAJB6 | Acts as a molecular chaperone for various cellular processes. |
| IFNGR2 | Participates in the JAK2-Signal transducer and activators of transcription proteins (STAT) signaling pathway (JAK2-STAT) via signal transduction. |
| NFATC4 | The product of this gene induces the expression of cytokine genes in T cells. |
| STAT1 | Participate in a variety of immune pathways and play an important role in mediating type I interferon (IFN-α/β) and type II interferon (IFN-γ). |

**Supplementary Figure S1:** DNA methylation level (%) of different CpG sites in GDM and Control group.

**Notes:** Little red triangle means the methylation levels of samples and the three black lines means the mean±95%CI.

**Reference**

1. Enquobahrie DA, Moore A, Muhie S, et al. Early Pregnancy Maternal Blood DNA Methylation in Repeat Pregnancies and Change in Gestational Diabetes Mellitus Status-A Pilot Study. *Reprod Sci* 2015;22(7):904-10. doi: 10.1177/1933719115570903 [published Online First: 2015/02/14]

2. Wu P, Farrell WE, Haworth KE, et al. Maternal genome-wide DNA methylation profiling in gestational diabetes shows distinctive disease-associated changes relative to matched healthy pregnancies. *Epigenetics* 2018;13(2):122-28. doi: 10.1080/15592294.2016.1166321 [published Online First: 2016/03/29]

3. de la Garza AL, Etxeberria U, Palacios-Ortega S, et al. Modulation of hyperglycemia and TNFalpha-mediated inflammation by helichrysum and grapefruit extracts in diabetic db/db mice. *Food Funct* 2014;5(9):2120-8. doi: 10.1039/c4fo00154k [published Online First: 2014/07/09]

4. Naidoo V, Naidoo M, Ghai M. Cell- and tissue-specific epigenetic changes associated with chronic inflammation in insulin resistance and type 2 diabetes mellitus. *Scandinavian journal of immunology* 2018;88(6):e12723. doi: 10.1111/sji.12723 [published Online First: 2018/12/28]

5. Kang J, Lee CN, Li HY, et al. Association of Interleukin-10 Methylation Levels With Gestational Diabetes in a Taiwanese Population. *Front Genet* 2018;9:222. doi: 10.3389/fgene.2018.00222 [published Online First: 2018/07/11]

6. Li E, Luo T, Wang Y. Identification of diagnostic biomarkers in patients with gestational diabetes mellitus based on transcriptome gene expression and methylation correlation analysis. *Reprod Biol Endocrinol* 2019;17(1):112. doi: 10.1186/s12958-019-0556-x [published Online First: 2019/12/29]
